# Investigation of opposition to diagnostic or therapeutic procedures in older people hospitalized in acute geriatric services: the OPTAH pilot study protocol

**DOI:** 10.1186/s40814-020-00742-7

**Published:** 2020-12-14

**Authors:** Thomas Tannou, Hélène Trimaille, Florence Mathieu-Nicot, Séverine Koeberle, Régis Aubry, Aurélie Godard-Marceau

**Affiliations:** 1 Geriatrics Department, University Hospital of Besançon, Besançon, France; 2 Equipe “Ethique et Progrès Médical” Inserm, CIC 1431, Centre d’Investigation Clinique, University Hospital of Besançon, Besançon, France; 3 EA 481 Neurosciences, UBFC, Besançon, France; 4 EA 3188 Laboratoire de psychologie, UBFC, Besançon, France

**Keywords:** Geriatrics, Decision-making, Opposition to treatment, Qualitative study, Epidemiology, Protocol

## Abstract

**Background:**

Shared decision-making is a process that involves collaborative discussions between a patient and a care team to ensure informed healthcare decisions. This process becomes more complex when the older person’s decision-making capacities are affected. In these situations, surrogate decision-making processes are used to define a person-centered care plan. Despite these processes, the implementation of the care plan defined in the best interest of the patient may nevertheless be rejected by the patient, particularly in cases of neurocognitive disorders or delirium. This concept of opposition and/or refusal is frequently used in research. This is not yet well understood in the medical literature, and there is a lack of consensus on its definition. We, therefore, explored this concept by defining opposition to diagnostic or therapeutic proposals.

**Method:**

Our pilot study protocol is based on a mixed methodology (epidemiological and qualitative research) to quantify this phenomenon, validate the proposed definition, and explore its core elements. Opposition and refusal of care will be quantified, and semi-structured interviews will be conducted with patients, their relatives, and referring carers. Multidisciplinary meetings that will be associated with these situations will also be observed and analyzed. Methodological approaches that can be used to explore opposition and refusal of care in a scientific, reproducible framework are presented. This methodology considers the specificities of the geriatric, polypathological population with neurocognitive disorders.

**Discussion:**

Opposition and refusal of care are key concepts in clinical research on ethics, particularly in the geriatric field. These concepts are frequently mentioned in studies involving older patients but have not been specifically defined or studied. This study would undoubtedly lead to greater awareness among professional caregivers and relatives of the significance of such opposition, and more respectful interactions in these complex hospitalization cases.

**Trial registration:**

ClinicalTrial.gov, NCT03373838. Registered on 14 December 2017.

## Background

Projections of population aging show that the proportion of people over 60 years of age in developed countries will increase from 20 to 30% between 2005 and 2050 [1]. Population aging is explained by the age pyramid but also by the increase in life expectancy. A particular effect associated with population aging is the increase in the use of health systems by people with multiple chronic diseases [2]. As we age, the management of polypathology becomes more complex; frailty gradually sets in, and various functional, psychological, social, and ethical issues need to be taken into account [3]. The number of older dependent people is also increasing with population aging [4]. Concomitantly, hospital use by older people with polypathology and dependence is high. Indeed, nearly 40% of patients over 75 years of age are hospitalized following a visit to the emergency department [5]. During these hospitalizations, many health-related decisions are made.

Health decisions must be based on the free and informed consent of the person involved. This principle, although already in use, was first universally recognized in the Nuremberg Code in 1947. It was later developed and refined, concerning both biomedical research and the practice of medicine. In this respect, various laws refer to patients’ freedom of decision, such as Article L. 1111-4 of the *French Public Health Code*: *No medical act or treatment may be performed without the free and informed consent of the person and such consent may be withdrawn at any time* [6, 7]*.* Thus, during hospitalization, each medical procedure is subject to the patient’s consent, if he/she can consent [8, 9]. When the person is no longer able to express his or her will, that is to say, based on the Mental Capacity Act (UK)—Section 3, that the person is no longer able: (a) to understand the information relevant to the decision, (b) to retain that information, (c) to use or weigh that information as part of the process of making the decision, or (d) to communicate his decision (whether by talking, using sign language or any other means), then surrogate decision-making should be made.

This surrogate decision-making includes substituted (trusted person) or delayed consent (advance directives) in a deliberative process to choose the most appropriate care option for the incapable person. Decision support frameworks are then based on the person-centered care approach to ensure the primacy of individuals’ health and life goals in their care planning and their actual care [10].

Despite changes in decision-making capacities associated with aging (see systematic review on this subject [11]), the older patient will make his/her choice according to rational and irrational information, and the stability of this choice overtime is expected [12]. Several tools have been developed [8, 13] to facilitate this assessment by health professionals, particularly in cases where the ability to consent is uncertain. Indeed, older patients are more likely to present alterations in decision-making processes due to neurocognitive disorders (even in mild stages) that create barriers to enlightened choices [14–19]. This is particularly the case during delirium [20].

Yet, routine care situations and low-risk procedures are rarely presented as choices, even though the patient has the right to refuse them [21]. Existing clinical tools assessing the ability to consent (e.g., [8, 19]) are therefore not used in this context. Patient’s opposition in situations that are not perceived by health professionals as open-ended proposals may lead to conflictual situations, in particular, if these situations are seen, by professionals, to be respectful of patient-centered care approach (e.g., antibiotics administration and blood test to treat sepsis and being able to discharge the patient to home). When a patient’s choice goes against medical strategies, the capacity to consent is often contested, as health professionals are reluctant to accept the risks involved [22]. Indeed, this situation opposes two contradictory obligations: on the one hand, the need to respect the person’s right to make a decision, according to the principle of autonomy, and on the other hand, the need to protect individuals, according to the principle of beneficence [23]. This leads to the need to reconsider the therapeutic objectives or the approach implemented and/or to agree collegially to override this opposition in the name of the patient’s best interest. To do this, it is necessary to ensure that the patient’s will and life project are understood, as much as possible, and to combine the skills of professionals to propose a care project adapted to the situation and centered on the person [24]. Nevertheless, despite these elements guaranteeing ethical respect for the approach decided by derogation, this may still lead carers to use procedures for which the patient is opposed.

However, even though the question of oppositional behaviors has been at the heart of many conceptual works in the field of geronto-psychiatry for more than 30 years, its study in clinical situations, and in particular during short hospital stays, remains rare. Indeed, conceptual approaches have used the prism of person-centered care to address the limitations of the care plan. In acute care, older patients may behave in opposition to medical strategies, even though the objective deployed corresponds to the patient’s previous personalized care plan. The technical nature of the treatment may lead the patient to oppose its implementation, particularly in cases of neurocognitive disorders. As such, the question of patients’ opposition to diagnostic or therapeutic proposals is a recurring issue faced by medical and paramedical teams. How can we explore the meaning of this opposition, when, paradoxically, it does not seem nameable, at first glance, by the patient? It is essential for carers and doctors, but also for relatives, who are often at the forefront of delegated decision-making processes [25], to obtain explanatory elements on this question to better position themselves and find the right balance between patients’ right to autonomy and the need to intervene to preserve his/her health.

## Hypotheses

The hospitalization of polypathological seniors represents a break in their life course. The technicity of medical care is generally engaged in a systematic way and often contributes to acute decompensation [26]. However, even though some medical interventions (e.g., insertion of a venous catheter for hydration or antibiotic therapy, additional medications, bandaging, biological check-ups) may seem, in the best interest of the patient, acceptable and necessary according to caregivers, and as such, compatible with internationally recognized legal and moral safeguards, their implementation may lead to vehement or even violent opposition if they exceed patient’s tolerance and dependence threshold [27]. Another hypothesis is that patients may not feel that their best interest is considered in recommended procedures. These patients may thus oppose diagnostic or therapeutic procedures to demonstrate their need or capacity to control their situation. Finally, the question of anosognosia and neurocognitive disorders raises the question of the meaning of treatment. Memory disorders may alter patients’ recollection of acute events once symptoms have been controlled, particularly if these events generated episodes of delirium.

A quantitative study seems necessary to quantify and better understand the parameters associated with situations involving opposition to diagnostic and therapeutic procedures. Although these events are often mentioned in medical literature, they are not documented or compiled in hospital records. Epidemiological studies must be associated with qualitative and comprehensive analyses of events to ensure an in-depth understanding of the opposition expressed by older patients. Is this opposition linked to a distorted and negative representation by patients of the proposed diagnostic or therapeutic procedure? In older people with a certain degree of delirium and/or cognitive deterioration, treatment may be perceived as a threat, intrusion, coercion, obstruction, violence, or even aggression. It is therefore conceivable that some intrusive procedures may be refused. Is this opposition an expression of a desire to control one’s own life until death? Is it not the ultimate expression of the existence of oneself, the expression of an ability to decide for and by oneself, in a world characterized by several constraints and dependence for all acts of daily life? Is this opposition expressing a feeling of indignity experienced in a societal context, or a reaction to ageist tendencies [28, 29] that can generate a sense of social uselessness among the older population? The qualitative component of the present study also contributes to the exploration of interactions between doctors, carers, and relatives confronted with patient opposition; the functioning of collegial meetings and decision-making methods are also addressed to better understand some consequences of the dilemma that arises in cases of opposition to diagnostic or treatment in older patients.

In order to answer these questions and verify the aforementioned hypotheses, it is necessary, in view of the specificities of opposition to medical care and target population (older people, including persons with neurocognitive disorders), to carry out a pilot study beforehand, with the following specific objectives:

- To verify the applicability of the proposed definition of opposition to medical care and its reproducibility
- Identify the proportion of patients expressing opposition to medical care according to the proposed definition
- Identify the key elements to be included in the qualitative interview, based on a preliminary interview grid.

## Methods

The present pilot study aims to investigate the situation of patients who are in opposition and to understand why they are opposing. The design is therefore prospective and monocentric. The protocol presented is based on the SPIRITS guidelines [30]. It was adapted in order to take into account the mixed methodology of the study and guidelines of pilot and feasibility studies [31, 32]. The objective is to explore participants’ positioning following diagnostic or therapeutic procedures that they declined. More specifically, characteristic components of these medical situations and arguments of the various protagonists were identified to understand the representations and symbolic functions linked to the opposition to medical acts. The study is divided into 2 parts:

- An epidemiological survey aiming to identify situations with opposition to diagnostic or therapeutic medical procedures in an acute geriatric ward
- A comprehensive study with a qualitative methodology based on semi-structured interviews that were conducted with carers or relatives involved in the aforementioned situations, to gain a more specific understanding of the factors involved in opposition to diagnostic or therapeutic procedures

### Definition of opposition

To date, there is no scientific consensual definition of opposition to treatment or diagnostic procedures. In the OPTAH study, we will define opposition to treatment or diagnostic procedures as follows:

1. The repeated (at least twice in a row, with at least two separate interlocutors) and explicit (verbal or physical) expression of an opposition. Oppositions expressed during the usual meetings between doctors and patients are not included in the analysis if they are part of a free and informed choice, even if they may concern a decision (acceptance or refusal) of a therapeutic proposal. Opposition behaviors concern either of the following:
  - Medication intake
  - Invasive diagnostic or therapeutic procedures (biological check-up, imaging, endoscopy, biopsy, etc.)
  - Removal by the patient of a venous, nasogastric, or urinary catheter
2. In the absence of consent or acceptance by the patient of similar treatments during the reporting period

It should be noted that we will not include in this definition non-medical care (meals, shower, massage, physiotherapy) if it does not have medical consequences, to avoid confounding factors and information gathering issues. Opposition to non-medical care deserves separate research studies. Thus, patients who oppose treatments or diagnostic procedures will be included in the study even though they otherwise accept non-medical care. Symmetrically, patients who do not oppose treatments and diagnostic examinations but who refuse care (meals, shower, massage, etc.) will not be included in the study unless their refusal (e.g., to drink) may lead to the development of a medical pathology (e.g., functional kidney failure due to dehydration) or medical consequences, for which they refuse medical investigation or treatment.

### Study objectives

- Epidemiological component: In the epidemiological survey, the objective will be to identify and establish the frequency of situations in which patients will express opposition to treatment or diagnostic procedures. The data will be obtained in the acute geriatric ward of a French university hospital, over 1 year.
- Comprehensive component: During the semi-structured interviews with carers and relatives involved in the epidemiological component, the objective will be to identify, describe, and understand the meaning of the opposition to treatment or diagnostic procedures expressed by patients included in the study (see below).

### Population and duration of the study

Patients over 75 years of age who will oppose diagnostic or therapeutic procedures during hospitalization and who will be hospitalized in the acute geriatric ward of a French university hospital will be included in this study. The acute geriatric care department comprises 54 hospitalization beds spread over 2 sections, including 6 geriatric intensive care beds. Medical care is provided with a ratio of one doctor and one intern for every 12 patients in conventional beds and 1 for every 6 patients in the geriatric intensive care unit.

To obtain an exhaustive overview of this population, the epidemiological data collection will involve a standardized survey that includes questions on descriptive characteristics of patients, which will be reported at the time of admission and during a follow-up in geriatric hospital services (collection of medical history, treatments, medico-social data, standardized geriatric assessment). A 1-year recruitment period will be used in this study. Among the patients that will present opposition to treatment or diagnostic procedures, we will select patients that can be interviewed (i.e., without cognitive impairment or with mild to moderate neurocognitive disorders which are not predominantly phasic) for the comprehensive (qualitative) component of this study. It is anticipated that recruitment for interviews could be difficult in this population, and the inclusion period for the second component is 2.5 years. The epidemiological data of patients included in the second year of the comprehensive component will be added to the initial data to allow comparisons between the groups participating in the two components. We will thus verify that for the population included in the comprehensive component, the characteristics of the opposition are similar to those of the overall study population.

The total duration of the study will therefore be 30 months.

#### Inclusion criteria

**Fig. 1:**
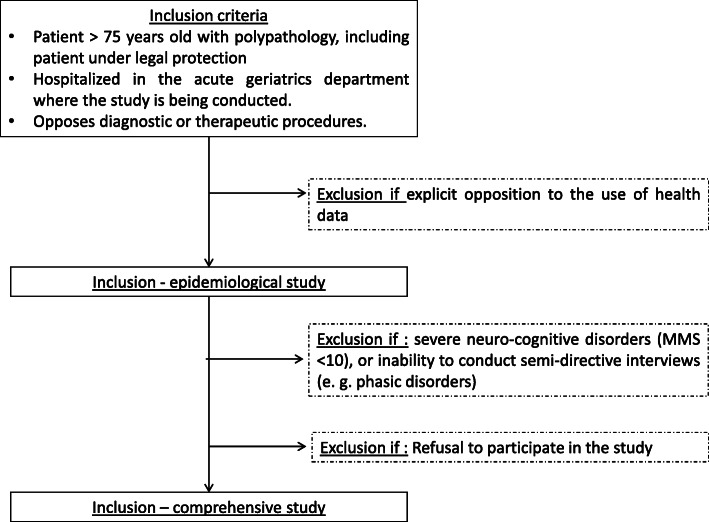
Inclusion flow diagram

In the epidemiological study, participants will be patients that meet the following inclusion criteria (see Fig. 1):

- Being hospitalized in the acute geriatrics department of a French university hospital where the study is conducted
- Having expressed opposition to diagnostic or therapeutic procedures, as defined above
- Not expressly objecting to the collection of personal data

For the comprehensive (qualitative) part, the inclusion criteria include patients, carers, and relatives who:

- Signed an informed consent form
- Can participate in a semi-directive interview with the researcher (the Mini-Mental States > 10, no phasic disorders)

Patients under legal protection are considered vulnerable. Their dependency is higher, and fundamental decisions concerning their care are most often taken by substitutes. However, these patients can still express opposition to care in various ways. Since they are under legal protection, listening to the expression of their will allows a better understanding of their precarious condition. As such, their integration into the research protocol is necessary. A specific information and consent form will be presented to the patient and guardian, and both consents will be obtained.

As this study will be a pilot study, we will include patients with all forms of cognitive status. More specific studies should be developed in the future.

#### Exclusion criteria

For the epidemiological part, patients will be excluded if they explicitly oppose the use of their health data.

For the comprehensive (qualitative) part, the following exclusion criteria will be used:

- Patient with severe neuro-cognitive disorders [33, 34] already known or diagnosed at service entry (MMS < 10) or inability to take part in a semi-directive interview (e.g., phasic disorders)
- Opposition to care that is not explicit or provided by a third party
- Refusal to participate in the study

### Experimental planning (see Fig. 2)

The time of the opposition, as defined above, corresponds to D0.

**Fig. 2:**
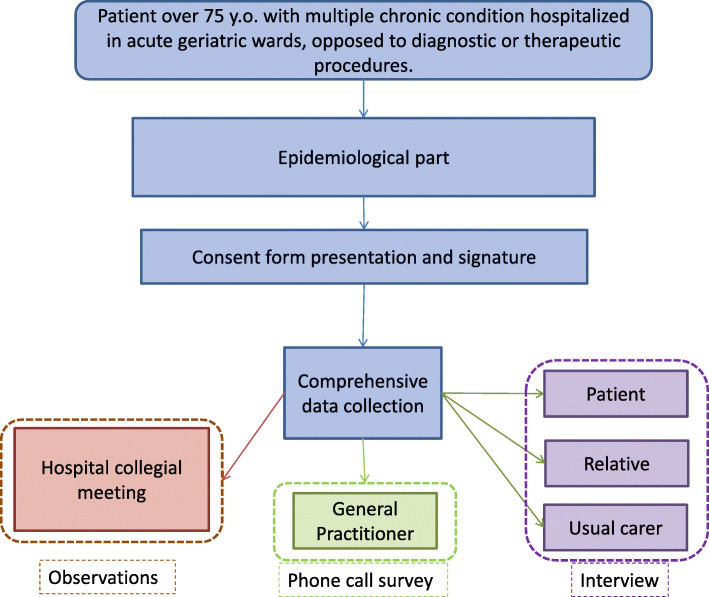
Experimental planning

#### Epidemiological component

When patients will present opposition to a diagnostic or therapeutic procedure, they will be included in the epidemiological component of this study by the attending physician. The physician will complete a data collection form to initiate the patient’s participation in the study. This form covers the patient’s general demographic and medico-social data. It focuses on the information related to the opposition that had occurred (drugs, procedures) and on the circumstances of the opposition (e.g., organ failure, sepsis, delirium). This survey will also be completed at discharge, to describe the outcomes resulting from this opposition (respect or not for the patient’s position, healthcare strategy, mortality).

The data collected are detailed below, in the “Data analysis” section.

#### Comprehensive part

Among patients presenting an opposition, we will select about ten situations that will be included in the comprehensive part of the study, to explore these situations through qualitative methodological tools (interviews, surveys, and observations). All epidemiological data will also be collected for these patients.

Thus, a comprehensive data collection will be conducted as follows:

- A semi-directed interview will be carried out with the patient, a relative designated by the patient, and the usual carer at home or the healthcare institution. These interviews (3 per situation) will be conducted by a researcher, a psychologist, or a sociologist (the complementarity of the two scientific disciplines allows triangulation of data collected in the various interviews), according to a pre-established interview framework (see Table 1).
- Medical questionnaire for the general practitioner (1 per situation). The qualitative medical form will be completed by the investigating physician during a telephone call with the family physician.
- As soon as the opposite situation leads to difficulties in medical care, and as soon as the setting up of collegial meetings will be organized, these meetings (at least 1 per situation) will also be attended by a psychologist and a sociologist of the research team. They will use an observation matrix to describe the content of collegial meetings, as observers (non-participants) in the discussion*.*

**Table 1:** Themes to be used during semi-structured interviews

Themes to be used during semi-structured interviews	
**History of care**	History of hospitalization
	Medical decisions
**Relationship with the caregiver**	Precedence of medical decisions
	Relationship with health professionals
**Home living/medicalization**	Relatives
	Home carer
	General practitioner
	Dependency
**Representation**	Previous experience with the illness, hospitalizations, treatments, operations

About 10 patients will be included in the comprehensive study. The sample could be re-evaluated if there is no data saturation, or if there are some difficulties to collect data because of patients’ cognitive status.

The verbatims will be analyzed according to the principles of the grounded theory (see below).

### Data collection strategies

#### Recruitment

This study aims to investigate opposition situations. To make a rigorous analysis, it seems necessary to do an exhaustive census of opposition situations. In that way, we will quantify the number of situations in which there is opposition to diagnostic or therapeutic procedures handled in the department for 1 year.

Given the population and the subject of this study, it is foreseeable that opposition to participation will occur, even though our focus on the refusal and thus the interest and respect for the person’s will can facilitate exchanges. This concern justifies the extension of the comprehensive study for an additional year and a half.

#### Epidemiological component

The medical and medico-social data used for the quantitative component will be compiled in a standard electronic form. The data will be collected during the consultation of the patients’ usual medical records including H24 daily notes from medical, nurses, and auxiliary nurses.

To characterize the population under study, the following data will be used:

- Demographic characteristics of patients (age, gender)
- Medical data of patients that are generally collected at the time of their admission (history, usual medications, nutritional, cognitive, motor, thymic status)
- Medico-social data relevant to the standardized geriatric assessment usually carried out in the service (capacity to perform activities of daily living, instrumental activities of daily living, place, way of life, presence of family and relatives, home-support workers)

To define oppositional behaviors, the following data will be collected:

- Circumstances of the opposition (who notices it, when, who reports it in the care record, recurrence of the event)
- Patient’s oppositional attitude (oral argumentation, physical opposition, catheter removal, type, duration)
- The target of opposition (single clinical element or overall attitude of catheter withdrawal and refusal of the procedure, taking medication, etc.), medication (molecule, route of administration), and/or material (venous, nasogastric, or urinary catheter) that triggered patients’ opposition
- Medical circumstances (organ failure, sepsis, delirium, etc.) surrounding the refusal of diagnostic or therapeutic procedures

To better understand the consequences of the opposition behavior, the following data will be collected:

- Medical decisions about the diagnosis or therapeutic strategies (continuation despite patient’s opposition or acceptance of patient’s opposition and change of medical orientation)
- The organization of the collegial meeting and the reasons involved
- Patients’ outcomes following the opposition behavior (discharge, transfer to another unit, death)

#### Comprehensive component

##### Semi-structured interviews

Semi-structured interviews allow the respondent to speak while the interview is focused on the subject of the study (the opposition) [33]. They are conducted using an interview matrix that presents the topics that will be discussed with the interviewee. When the topics are determined in advance, their order and presentation format are decided by the interviewer. The researcher has a series of relatively open-ended guiding questions directly based on the themes to be covered (see Table 1) for which he/she wishes to obtain information. Semi-directed interview guides are developed and tested in consultation between the investigating physician and researchers involved in the project. They are adapted and improved during the study to explore all the themes that are essential to understand the situation.

During an interview, the relationship between the interviewer and the subject is based on trust, respect, and empathy. The subject must be comfortable, knowing that the interview is a privileged moment, so that he/she can express his/her experience, his/her journey, and his/her representations. He/she must perceive that the researcher is there to listen to him/her. The researcher’s listening position is essential to allow the respondent to tell his/her subjective experience and to move away from what he/she can socially consider as expected by the researcher [34].

Semi-directed interviews will be recorded with a voice-recorder and fully transcribed anonymously and confidentially. In the information note and consent, the patient, family member, and caregiver will be informed of this modality.

The objective of the semi-directive interview with the patient is to understand the meaning or justification for this opposition. The objective of the meeting with the family member and the usual caregiver (institutional caregivers, if applicable, or primary caregiver at home) is to understand the positioning of this opposition with the patient’s previous expressions, to collect their analysis or interpretation, and to cross-reference the elements reported by the patient, family members, and caregivers.

##### Medical survey

General practitioners are a research population that is difficult to reach in qualitative research because of time constraints resulting from their practice. The telephone questionnaire completed with the family physician will take into account professional requirements and their limited availability and allows to collect information on the patient’s prior positioning concerning any limitation of care. The family physician will report his/her analysis or interpretation of the patient’s positioning, which will be cross-referenced with other elements collected during interviews with the patient, his/her relatives, and hospital carers. Differences in perceptions will be used to bring out a more comprehensive meaning of the opposition that will be reported for each patient.

The use of qualitative telephone surveys (structured interviews) makes it possible to partially overcome availability constraints, and thus integrate into this study the point of view of the general practitioner, which is essential for the overall understanding of the patient’s positioning in opposition to care, especially since phone exchanges are usual in these circumstances, and primary practitioners appreciate when they are included in the discussion surrounding these situations.

##### Observation of hospital collegial meetings

It is during collegial discussions that decisions are made in response to patients’ opposition to care. During these discussions, and as part of a team dynamic, each caregiver expresses his/her opinion about the patient’s situation and explains the possible justifications for the limitation of diagnostic or therapeutic procedures. The objectives of collegial meetings are to have an overview of the patient’s positioning and discuss the different points of view. The objective of observing a hospital meeting concerning the complex management of the opposition to care is to evaluate the significance given to the patient’s positioning in acute contexts by medical and paramedical teams. Indeed, this opposition potentially challenges the foundations of hospital-based, active, and performance-based medicine.

The observation will be conducted by a researcher (psychologist or sociologist). In order not to restrict exchanges, and thus facilitate the free expression of the teams, it is decided not to record these exchanges, and notes will be taken by the researchers to record the dynamics of the exchanges, the positions, and the questions raised by caregivers.

### Data analysis

#### Statistical analysis of epidemiological data

Analyses for the quantitative component are based on the data that will be extracted from medical records and descriptive statistics calculated based on this data.

The data of the reference population for statistics on prevalence and comparisons with the general population will be determined according to results obtained in a survey of professional practices conducted within the same geriatrics service. An anonymized database was therefore created to compile the medical and socio-demographic data of 60 patients that were seen in this ward for 1 month. These patients are representative of the population that receives health services in this hospital, which allows comparisons with the study group.

A descriptive analysis of the data will be conducted. Patient characteristics will be described using mean and standard deviation (SD) or median and interquartile range (IQ) for quantitative variables and using counts and percentages for qualitative variables. Variables will be compared using either the chi-square test or Student’s test. A difference with a *p* less than 0.05 will be considered significant. The R software will be used for these analyses.

#### Qualitative methodology

Interviews will be conducted until saturation of data and concepts is obtained, i.e., when the inclusion of a new participant no longer provides additional information to the investigation. This is an interactive process where new data are integrated progressively with the analysis of previous data. This integration provides new insights into the work that has been done and helps to plan the next interviews, when they are necessary. If the newly collected data does not bring new information, i.e., when the categories include all the elements of the phenomenon, and the links between the categories are established and validated, data saturation is reached [35].

All interviews and observations collected during team meetings will be transcribed or narrated anonymously and confidentially. Thus, the names of persons and places mentioned in the interview are not included in the transcripts.

It is considered that a rigorous analysis of interviews is only possible if the content of their recording is transcribed in writing. As some written documents will be consulted during the interview, the full transcription includes all verbal, para-verbal, and non-verbal information presented by the interviewee and the interviewer. All statements in the transcription are in the order in which they were stated. Repetitions and speech errors will not be removed from the transcript. Punctuation rules which are essential for the readability of the text (comma, endpoint, suspension point) will, however, be respected. The transcriber will set the rules for transferring from the verbal to the written register in a conventional way, and he/she will comply with them for all research interviews.

Para-verbal and non-verbal information, i.e., non-linguistic features that accompany speech, will be included in the full transcription. Para-verbal features of speech include voice inflections, accents, and whispers, as well as speech flow, hesitations, pauses, and silences. Non-verbal elements that accompanied and punctuated the speech will also be noted: smiles, laughter, pouting, crying, etc. These various gestures and mimics contribute to the subjective, conscious, or unconscious expression of feelings, opinions, and judgments of the interviewee.

The analysis of transcriptions will be carried out by the two researchers who will conduct the interviews. The researchers will analyze raw data to extract themes. They will take an inductive approach to identify patterns, highlighting topics that repeatedly emerge, characterizing them, and then organizing them into themes, which they will review and discuss to reduce the likelihood of personal bias and to ensure analytic robustness. Finally, they will summarize the themes to demonstrate the pattern of their findings. These analyses will then be collectively discussed by the multidisciplinary research team involved in the project, which included psychologists, sociologists, philosophers, and physicians. This interdisciplinarity will allow for a cross-referenced analysis, to reduce the subjectivity potentially associated with the researcher and his/her field of expertise and support the validity of the results.

The analysis of qualitative data (semi-structured interviews and observation notes) is consistent with the principles of the grounded theory: the data collected is used to construct an explanatory theory answering the initial question [36, 37]. Thematic analysis is carried out to form a thematic tree. The interpretation of this thematic tree allows for the emergence of concepts that constitute the comprehensive theoretical model for the situations under study [38].

## Discussion

Opposition and refusal of care are key concepts in clinical and ethical research, especially in older people. These concepts are frequently mentioned in studies involving older patients but have not been specifically defined. Our research protocol aims to investigate this field, through a pilot study using a reproducible methodology which is adapted to in-hospital services for short geriatric stays. Nevertheless, as this is a pilot study, it has the limitation of exploring opposition based on medical traces and comprehensive interviews carried out after this behavior occurs. In the long term, an exploration using ethnographic techniques would be preferable, based on the exploratory results of this pilot study.

Of course, the patient is free to make his/her medical decisions and the health professional has a responsibility to offer him various options and to accompany any decision made by the patient in the best possible way. Nevertheless, what justifies our study is the necessity of a better understanding, in the particular situation of neurocognitive diseases, of patients’ decision-making in cases where these decisions are not necessarily well informed due to cognitive disorders. It is essential to explore whether the perceived opposition behavior is enlightened, and requires a repositioning of the healthcare team to best support the patient’s wishes, or whether there is a lack of understanding of the situation, and therefore an uninformed opposition which must then impose a decision by derogation in accordance with ethical rules, following multidisciplinary meetings.

Indeed, the communication of a solid description and a thorough understanding of the opposition of hospitalized seniors to diagnostic and therapeutic medical procedures will provide professionals with results that can be used in their current practice related to the care and support of older people, while respecting their autonomy and wishes, to improve patient-centered care approach and to help managing patient’s best interest surrogate decision-making.

As such, the results of the present study can be used to develop training modules that will contribute to the prevention and support of situations involving opposition to diagnostic or therapeutic procedures.

## Study status

The recruitment period began on 1 January 2018 and therefore extends, for the comprehensive part, until 30 June 2020. After 20 months of data collection, the quantitative collection comprised 33 situations in the epidemiological component, of which 5 situations were included in the qualitative component (interviews).

## Data Availability

The datasets used and/or analyzed during the current study are available and can be provided by the corresponding author on reasonable request.

## Abbreviations

MMSE: Mini-Mental Status Examination
OPTAH: Opposition to diagnostic or therapeutic procedures in older people hospitalized in acute geriatric services
